# Characteristics of 24 SARS-CoV-2-Sequenced Reinfection Cases in a Tertiary Hospital in Spain

**DOI:** 10.3389/fmicb.2022.876409

**Published:** 2022-05-26

**Authors:** Blanca Borras-Bermejo, Maria Piñana, Cristina Andrés, Ricardo Zules, Alejandra González-Sánchez, Juliana Esperalba, Oleguer Parés-Badell, Damir García-Cehic, Ariadna Rando, Carolina Campos, Maria Gema Codina, Maria Carmen Martín, Carla Castillo, Karen García-Comuñas, Rodrigo Vásquez-Mercado, Reginald Martins-Martins, Sergi Colomer-Castell, Tomàs Pumarola, Magda Campins, Josep Quer, Andrés Antón

**Affiliations:** ^1^Department of Preventive Medicine and Epidemiology, Vall d’Hebron Research Institute, Universitat Autònoma de Barcelona, Barcelona, Spain; ^2^Respiratory Viruses Unit, Virology Section, Microbiology Department, Vall d’Hebron Institut de Recerca (VHIR), Vall d’Hebron Barcelona Hospital Campus, Vall d’Hebron Hospital Universitari, Barcelona, Spain; ^3^CIBERINFEC, ISCIII-CIBER de Enfermedades Infecciosas, Instituto de Salud Carlos III, Madrid, Spain; ^4^Liver Diseases-Viral Hepatitis, Liver Unit, Vall d’Hebron Institut de Recerca, Barcelona, Spain; ^5^Centro de Investigación Biomédica en Red de Enfermedades Hepáticas y Digestivas, Instituto de Salud Carlos III, Madrid, Spain

**Keywords:** SARS-CoV-2, COVID-19, reinfection, whole-genome sequencing, clinical features

## Abstract

**Background:**

Since the emergence of severe acute respiratory syndrome coronavirus 2 (SARS-CoV-2), the main concern is whether reinfections are possible, and which are the associated risk factors. This study aims to describe the clinical and molecular characteristics of 24 sequence-confirmed reinfection SARS-CoV-2 cases over 1 year in Barcelona (Catalonia, Spain).

**Methods:**

Patients with > 45 days between two positive PCR tests regardless of symptoms and negative tests between episodes were initially considered as suspected reinfection cases from November 2020 to May 2021. Whole-genome sequencing (WGS) was performed to confirm genetic differences between consensus sequences and for phylogenetic studies based on PANGOLIN nomenclature. Reinfections were confirmed by the number of mutations, change in lineage, or epidemiological criteria.

**Results:**

From 39 reported suspected reinfection cases, complete viral genomes could be sequenced from both episodes of 24 patients, all were confirmed as true reinfections. With a median age of 44 years (interquartile range [IQR] 32–65), 66% were women and 58% were healthcare workers (HCWs). The median days between episodes were 122 (IQR 72–199), occurring one-third within 3 months. Reinfection episodes were frequently asymptomatic and less severe than primary infections. The absence of seroconversion was associated with symptomatic reinfections. Only one case was reinfected with a variant of concern (VOC).

**Conclusion:**

Severe acute respiratory syndrome coronavirus 2 reinfections can occur in a shorter time than previously reported and are mainly found in immunocompetent patients. Surveillance through WGS is useful to identify viral mutations associated with immune evasion.

## Introduction

In December 2019, an outbreak of severe acute respiratory disease caused by a new coronavirus was emerged in Wuhan, China. The disease, caused by severe acute respiratory syndrome coronavirus 2 (SARS-CoV-2) and named Coronavirus Disease-19 (COVID-19), was finally declared as a global pandemic by the World Health Organization on 11 March 2020. SARS-CoV-2 is an enveloped, single-stranded, positive-sensed RNA virus. Its 30 kb genome encodes for four major structural proteins, which are as follows: Spike (S), Envelope (E), Membrane (M), and Nucleocapsid protein (N); 16 non-structural proteins (nsp1–16) are encoded by the open reading frame (ORF) 1ab and six accessory proteins (ORF 3a, 6, 7a, 7b, 8, and 10) (Wu et al., 2020).

Since the beginning of the SARS-CoV-2 pandemic, more than 413 million people have been infected with more than 5 million deaths. One of the main concerns for public health is whether reinfection is possible despite the immune response elicited after the primary infection. Large studies suggest that this is an uncommon event, as natural immunity decreases the risk for reinfection (Hansen et al., 2021). Only a few cases of reinfection confirmed by sequencing have been reported in the literature (European Centre for Disease Prevention and Control, 2020), and scarce data are available regarding risk factors.

This study is aimed to describe the characteristics of the first 24 sequence-confirmed cases of reinfection detected from March 2020 to February 2021 in a tertiary hospital laboratory in Barcelona, Spain.

## Materials and Methods

### Sample Collection

From November 2020 to May 2021, upper and lower respiratory specimens were received for the laboratory confirmation of SARS-CoV-2 from children and adults accomplishing the World Health Organization case definition criteria of SARS-CoV-2 infection (World Health Organization, 2020), who were attended at the Hospital Universitari Vall d’Hebron (HUVH) or the Primary Care Centers of its influence area. Serums were also received from some patients for immunoglobulin G (IgG) and immunoglobulin G (IgM) detection.

### Case Definition of Reinfection

A suspected case of SARS-CoV-2 reinfection was defined as a patient with two SARS-CoV-2 positive PCR tests separated at least 45 days (based on the minimum interval described for reinfection according to The European Center for Disease Prevention and Control [ECDC]) (European Centre for Disease Prevention and Control, 2020), regardless of the presence of symptoms and the existence of a negative test between episodes. Cases should have available specimens from each episode for whole-genome sequencing (WGS) as inclusion criteria.

Cases were confirmed as true reinfections when at least one of the following criteria was accomplished: (a) a change of lineage in the second episode versus the first one; (b) the number of acquired nucleotide mutations was similar to or higher than the expected to happen during the time period between episodes according to the evolution rate estimated by Nextstrain (2 mutations per month approximately) (Hadfield et al., 2018); or (c) the presence of new compatible COVID-19 symptoms in the second episode or a history of recent contact with an infected person.

Suspected reinfection cases were not collected by systematic surveillance but reported by hospital staff to the laboratory.

### Detection of SARS-CoV-2

Detection of SARS-CoV-2 was carried out by non-commercial (2019-nCoV CDC PCR Panel and One-Step RT-PCR Kit, Qiagen, Germany), commercial real-time reverse transcription-polymerase chain reaction (RT-PCR) assays (Allplex™ 2019-nCoV Assay, Seegene; Cobas^®^ SARS-CoV-2 Test, Roche Diagnostics; and Xpert Xpress SARS-CoV-2 test, Cepheid), or transcription-mediated amplification (TMA)-based assays (Procleix SARS-CoV-2, Grifols; Aptima SARS-CoV-2, Hologic Inc. MA, United States), depending on the availability of the products over time. All assays were performed following the instructions of manufacturers. For RT-PCR assays requiring nucleic acids extraction, NUCLISENS easyMAG (bioMérieux, Marcy l’Etoile, France) and Microlab STARlet System (Hamilton, NV, United States) were used. Clinical SARS-CoV-2-positive specimens were kept frozen for further studies.

Serological testing was performed with Liaison SARS-CoV-2 S1/S2 IgG and IgM (Diasorin, Saluggia, Italy) or ELISA (Anti-SARS-CoV-2 ELISA IgG and IgA, Euroimmun, Lubeca, Germany), following the instructions of manufacturers. Assays were selected depending on the products’ availability.

### Whole-Genome Sequencing of SARS-CoV-2

Whole-genome sequencing of SARS-CoV-2 was performed following the ARTIC Network protocol^1^ with the V3 primer pools (Integrated DNA Technologies, IDT, IA, United States) for complete genome multiplex, overlapping amplification. Library preparation was performed with KAPA HyperPrep Kit (Roche Applied Science, Basilea, Switzerland) or Illumina DNA Prep (Illumina, CA, United States) depending on the product’s availability. Briefly, cDNA synthesis was performed with SuperScript IV reverse transcriptase (Invitrogen) and further PCR amplification for the two pools of ARTIC V3 primers with Q5 Hot Start High-Fidelity DNA Polymerase (New England BioLabs, MA, United States). PCR products from each sample were individually indexed using the SeqCap Adapter Kit A/B (Nimblegen, Roche, CA, United States) when using KAPA HyperPrep; or IDT^®^ for Illumina^®^ DNA/RNA UD Indexes Set A-D (384 IDX) (Illumina, CA, United States) when using Illumina DNA Prep. Finally, the products were added to the final pooled library tube, which, together with a 5% of PhiX internal DNA control (PhiX V3, Illumina, CA, United States), were loaded in a MiSeq Reagent Kit (600 cycles) v3 (Illumina, CA, United States) and sequenced using the MiSeq platform (Illumina, CA, United States).

### Bioinformatic Analyses

FastQ files generated in the MiSeq output were uploaded to BaseSpace Sequence Hub (Illumina, CA, United States) and analyzed with the DRAGEN COVID Lineage app for the Kmer-based detection of SARS-CoV-2, mapping of the passing filters’ reads to the reference genome (NC_045512 in GenBank), variant calling generation of a consensus genome sequence with a coverage threshold at least of 20×. Finally, Pangolin (version of 19 May 2021) was also run on those consensus sequences to determine the viral lineage. Mutations were reported using MEGA v6.0 software (Tamura et al., 2013), in comparison to the first episode sequence as the reference for each patient. Consensus sequences were uploaded to Global Initiative on Sharing All Influenza Data (GISAID) with accession numbers EPI_ISL_2284948, EPI_ISL_6595267-6595311, and EPI_ISL_6596214-6596215.

Random complete SARS-CoV-2 genomes sequenced at our hospital available at GISAID database (Elbe and Buckland-Merrett, 2017; Shu and McCauley, 2017) were selected to have a representative phylogenetic comparison of circulating SARS-CoV-2 strains in Barcelona, Spain. Maximum-likelihood phylogenetic analyses were inferred through TreeTime (Sagulenko et al., 2018).

### Clinical Features

Socio-demographic, clinical, and epidemiological characteristics for each episode were retrospectively reviewed from electronic medical records. Days between episodes were calculated according to the onset of symptoms that occurred before a positive test result.

Institutional Review Board approval (PR(AG)259/2020) was obtained from the hospital’s Clinical Research Ethics Committee.

### Statistical Analyses

Frequencies and percentages were used to describe categorical data. For continuous data, median and interquartile ranges (IQR) were calculated. For analysis of categorical data, chi-squared or Fisher’s exact test was performed. All computations were made in Excel and the R language (R Core Team, 2021). R: A language and environment for statistical computing and R Foundation for Statistical Computing (Vienna, Austria).

## Results

A total of 503,825 samples were received from November 2020 to May 2021, of which 54,101 (11%) were laboratory confirmed for SARS-CoV-2. Overall, 39 cases of suspected reinfections were reported to the laboratory (0.07% of positive samples). Samples of both episodes were recovered and were suitable for sequencing in 24 cases which all were confirmed as true reinfections according to the criteria above. The main characteristics of these 24 patients with COVID-19 reinfection are summarized in Table 1.

**TABLE 1:** Characteristics of 24 severe acute respiratory syndrome coronavirus 2 (SARS-CoV-2) reinfection cases (March 2020 to February 2021) Barcelona, Spain.

Feature	No. (%)
Age	44 (IQR 32–65)
Females	16 (66)
Health Care Workers	14 (58)
Hospital	6 (25)
Nursing home	8 (33)
Presence of comorbidities	10 (42)
Immunosuppression	2 (8)
**Reason for testing**	
**First episode**	
Suspected case	16 (67)
Screening	5 (21)
Contact tracing	1 (4)
**Second episode**	
Suspected case	6 (25)
Screening	13 (54)
Contact tracing	5 (21)
**Presence of symptoms**	
First episode	19 (80)
Second episode	14 (59)
**Hospitalization**	
First episode	6 (25)
Second episode	2 (8)

The median age was 44 years old (IQR 32–65). More than half of the cases happened in healthcare workers (HCWs) either from the hospital or nursing homes, whose median age was 38 (IQR 30–46), different from non-HCW cases, 68 (IQR 45–78). Among HCWs, 12 of 14 were women, compared to 4 of 10 non-HCWs. Comorbidities were present in 10 cases, and two were immunosuppressed.

**FIGURE 1:**
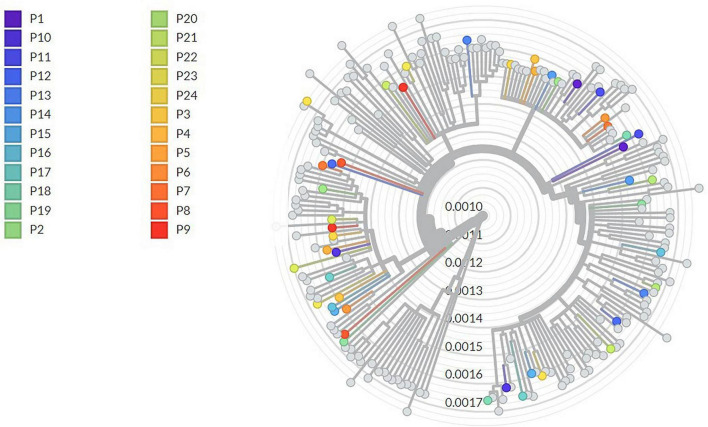
Phylogenetic tree of whole-genome sequences. Each patient has a color assigned, represented in the legend. The scale bar represents the mean number of substitutions per site.

Cases were confirmed as true reinfections due to a higher number of observed mutations than expected by intra-host evolution (P1–P14, P16–P19, P21, P23, and P24), the change of lineage (P3, P4, P8–P10, P12–P19, P21, P23, and P24), or due to clinical and epidemiologic criteria (presence of symptoms or recent contact with an infected person) (P20 and P22). Demographic, clinical, and molecular features of 24 sequenced suspected reinfections are presented in Table 2. WGSs of the 24 patients are represented in a phylogenetic tree together with other Spanish contemporary sequences in Figure 1.

**TABLE 2:** Clinical and molecular characteristics of 24 cases of severe acute respiratory syndrome coronavirus 2 (SARS-CoV-2) reinfection (March 2020 to February 2021) Barcelona, Spain.

Case	Age, sex, and status	Days between episodes	Lineage 1	Lineage 2	N° observed mutations (expected)	Spike mutation	Characteristics 1st episode and purpose of testing	Serology test result and days after infection	Characteristics 2nd episode and purpose of testing	Serology test result and days after reinfection
P1	73, male Comorbidities	75	B.1	B.1	24 (5)	V222A, H681P, V846A, F962L	Asymptomatic, Screening	ND	Asymptomatic, Screening	Positive, 21
P2	80, male Comorbidities	87	B.1	B.1	16 (6)	G614D, P681H, T716I, T719I, C1247F	Symptomatic, Hospitalized, Suspected case	Positive, 85	Asymptomatic, Screening	Positive, 232
P3	83, female Comorbidities	51	B.1.610	B	13 (3)	G614D	Asymptomatic, Contact tracing	Positive, 44	Asymptomatic, Screening	ND
P4	43, female NHW	106	B.1.610	B	5 (7)	A222V	Symptomatic, Hospitalized, Suspected case	ND	Symptomatic, Suspected case	Positive, 15
P5	47, female HCW Comorbidities	60	B.1	B.1	10 (4)	G614D, D1084H	Symptomatic, Suspected case	Positive, 60	Asymptomatic, Screening	Positive, 264
P6	31, female NHW	114	B.1	B.1	14 (8)	A222V	Symptomatic, Suspected case	Positive, 114	Symptomatic, Contact tracing	ND
P7	28, female HCW	119	B.1	B.1	10 (8)	–	Symptomatic, Suspected case	Positive,41	Symptomatic, Screening	ND
P8	29, female Pregnant	53	B.1.1	B.1.177	13 (4)	–	Symptomatic, Hospitalized, Suspected case	Positive, 45	Asymptomatic, Screening	Positive, 49
P9	26, female NHW	56	B.1.1	B.1	8 (4)	G614D	Symptomatic, Suspected case	ND	Symptomatic, Screening	ND
P10	30, female HCW	224	B.1	AA.1	19 (15)	A222V, D614G, A672V	Symptomatic, Suspected case	Negative, 48	Symptomatic, Suspected case	Positive,16
P11	29, female HCW	64	B.1	B.1	9 (4)	–	Symptomatic, Suspected case	Positive, 45	Asymptomatic, Screening	Positive, 38
P12	38, male HCW Controlled HIV	217	B.1.177.7	B.1.177	17 (15)	A222V, V701A, A682S	Symptomatic, Suspected case	Positive, 43	Symptomatic, Suspected case	Positive, 32
P13	37, male Comorbidities	125	B.1.1.406	B.1.177.32	22 (9)	A222V, V1251G	Asymptomatic, Screening	ND	Asymptomatic, Contact tracing	ND
P14	65, female NHW	220	B.1.610	B.1.177	16 (15)	L727I	Symptomatic, Screening	Negative, 97	Symptomatic, Suspected case	Positive, 32
P15	46, female NHW	125	B.1.177	B.1	9 (9)	–	Symptomatic, Screening	Positive,31	Asymptomatic, Screening	ND
P16	41, female	64	B.1.1	B.1.177	15 (4)	A222V	Symptomatic, Suspected case	Negative, 64	Symptomatic, Hospitalized, Suspected case	Positive, 13
P17	48, female NHW	159	B.1.177.31	B.1.177	24 (11)	A222V, D570A, P681H, T716I, N978K, D979Y	Asymptomatic, Contact tracing	Negative, 124	Symptomatic, Screening	ND
P18	86, male Comorbidities	291	B.1	B.1.177	22 (20)	T208M, A222V, R638T	Symptomatic, Hospitalized, Suspected case	Negative, 290	Symptomatic, Hospitalized, deceased. Suspected case	ND
P19	38, female NHW	163	B.1.177	B	17 (11)	D1118H	Symptomatic, Suspected case	Positive, 25	Symptomatic, Screening	Positive, 13
P20	66, male Comorbidities	193	B.1.177	B.1.177	7 (13)	–	Asymptomatic, Screening	ND	Symptomatic, Contact tracing	Positive, 18
P21	57, woman Renal transplant	223	B.1.1	B.1.177	19 (15)	A222V, P809S	Symptomatic, Hospitalized, Suspected case	Positive, 223	Symptomatic, Screening	Positive, 5
P22	33, female HCW	298	B.1	B.1	9 (20)	–	Symptomatic, Suspected Case	Negative, 82	Symptomatic, Contact tracing	Positive, 56
P23	45, female NHW	111	B.1.326	B	17 (8)	A570D, Y1084D	Symptomatic, Contact tracing	Positive, 26	Asymptomatic, Contact tracing	Positive, 7
P24	70, male	146	B.1.177	B.1.351	37 (10)	D215G, V222A, K417N, E484K, N501Y, Q654E, A701V, Y1163D, C1247F	Symptomatic, Hospitalized, Suspected case	Positive, 14	Asymptomatic, Screening	Positive, 1

**FIGURE 2:**
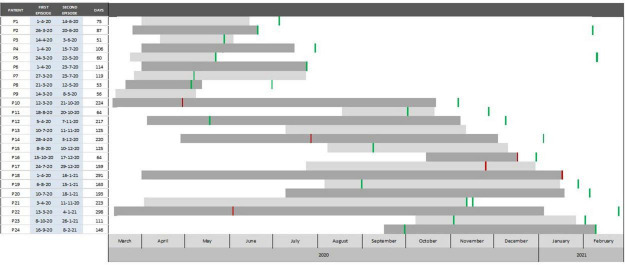
Temporary distribution of 24 severe acute respiratory syndrome coronavirus 2 (SARS-CoV-2) reinfection cases and days between episodes (March 2020 to February 2021) Barcelona, Spain. Red lines indicate the time of a negative serology, while green lines indicate the time of positive serology.

The time between episodes for each case and its distribution over time is represented in Figure 2. The median number of days between episodes were 122 (IQR 72–199), 51 days as the minimum period between episodes. The most frequent reason for testing during the first episode was due to compatible symptoms (*n* = 16), followed by screening (*n* = 5) and contact tracing (*n* = 3). Second episodes were detected mostly by screening (*n* = 13), followed by clinical suspicion (*n* = 6) and contact tracing (*n* = 5).

In the first episode, 19 (79%) patients manifested symptoms and 6 (25%) required hospitalization. After the first infection, 13 patients were detected to have specific anti-SARS-CoV-2 149 IgG antibodies, 6 were seronegative, and 5 were not tested for antibodies. In the second episode, 14 (58%) patients manifested symptoms, of which two (P16 and P18) required hospitalization and one was (P18) died. These two hospitalized cases presented symptoms in the first episode and did not seroconvert after it. Regarding the relation between symptoms and the presence of specific antibodies, among 13 cases that seroconverted after the first episode, five were symptomatic during the second, while all six cases without seroconversion had symptoms at reinfection. The presence of symptomatology in the reinfection episode was more frequent if there was no seroconversion following the first episode (*p* = 0.02). Upon the second episode, all tested patients (16/24) developed or maintained specific antibodies.

The most observed lineages were B.1 (*n* = 19; 40%) and B.1.177 (*n* = 12; 25%; Table 2). Other lineages included B.1.1, B.1.610, diverse descendants from B.1.177, and one variant of concern (VOC) B.1.351 (Beta variant), among others. The most prevalent mutations were in amino acid position 890 in nsp3, 54 in nsp6, 323 in nsp12, 222 and 614 in Spike, 220 in Nucleocapsid protein, and 30 in ORF10. Mutation in position 30 in ORF10 (V30L or L30V) was associated with the manifestation of symptoms in the second episode (*p* = 0.03). No relevant mutations were found in the two hospitalized patients.

## Discussion

This study reports clinical and molecular characteristics from 24 SARS-CoV-2 confirmed reinfections, supported by WGS or clinical and epidemiologic criteria. In all but one patient, reinfection happened with non-VOC lineages, similar to those circulating at the time of reinfection. Despite mutations were identified throughout the whole genome, amino acid changes within Spike were not overrepresented as could be expected.

Clinical characteristics of cases allowed us to identify two different main groups: young female HCWs either from the hospital or nursing homes, and patients over 50 years (half of them over 70 years), with 60% being men. Despite HCWs could be overrepresented in this study, the main characteristics of the patients in the present study reflect the well-known populations at high risk for severe illness as the elderly (Ko et al., 2021) and at increased risk of suffering COVID-19 due to increased exposure as HCWs (CDC COVID-19 Response Team, 2020). Thus, HCWs should continue strictly using routine prevention measures despite recovering from COVID-19, as intense exposure settings could increase the risk of reinfection. Except for one patient with controlled HIV and a renal transplant recipient, the rest of the reinfections occurred in immunocompetent patients, which has already been described (Choudhary et al., 2021).

The median days between episodes (122, IQR: 72–199) were similar to the reported in other studies (Choudhary et al., 2021; Qureshi et al., 2021). There is evidence supporting that reinfection is exceptional within 3 months after infection (Hansen et al., 2021). The Center for Disease Control and Prevention in the investigative criteria for suspected cases of reinfection includes those cases with a positive SARS-CoV-2 test ≥ 90 days after the first episode (Centers for Disease Control and Prevention [CDC], 2020). Nevertheless, this definition for suspected reinfection would have failed to identify one-third of our cases. Indeed, this study (with a 45 interval days criteria) describes four cases of reinfection occurring within 2 months. Other authors have reported confirmed reinfections by sequencing within 60 days (Abu-Raddad et al., 2020; Tillett et al., 2021), and one as low as in 19 days (Tang C. Y. et al., 2021). Recently, ECDC proposed a unified case definition for reinfection surveillance, lowering the days between episodes from 90 to 60 days, which will allow to better identification (European Centre for Disease Prevention and Control, 2021).

The presence of symptoms was more common during the first episode (80%) than during the reinfection (50%). Until June 2020, testing was limited to severe cases and symptomatic HCWs, which could have skewed our results. However, when focusing on cases whose first episode happened after June 2020 (P11, P13, P15, P16, P17, P19, P23, and P24), the same pattern is repeated, where the second episodes were more frequently asymptomatic. This decrease in the proportion of symptomatic cases was also observed in the SARS-CoV-2 immunity and reinfection evaluation (SIREN) study (Hall et al., 2021), where only 34% of individuals had symptoms at reinfection when compared with 79% at previous infection. This suggests that natural infection could prevent not only from reinfection but also from further symptomatic episodes and lower its severity.

The second episode was found to most commonly manifest with lower severity than the first one, as previously reported (Bongiovanni et al., 2021; Qureshi et al., 2021). However, reinfections can also manifest as severe diseases and should not be trivialized, as in our study, there were two hospitalizations (one was also admitted during the first episode), one of whom was fatal.

The high proportion of asymptomatic patients in the second episode could be underestimating the reinfection events, as they are only identified through contact tracing or screening strategies. Implementing systematic surveillance and other strategies as serial testing in high-risk populations would allow the identification of a higher number of asymptomatic cases contributing to transmission control.

Regarding seroconversion, it stands out that 13 patients had a positive antibody test after the first episode that did not prevent them from reinfection. On the other hand, negative sera tests after the first episode were more frequent to be found in symptomatic cases rather than in asymptomatic ones, suggesting that seroconversion could prevent further symptomatic episodes, along with reducing the risk of reinfection, as it is already described (Leidi et al., 2021).

Due to the presence of anti-SARS-CoV-2 IgG antibodies during the reinfection episode, five patients were neither placed under isolation precautions nor initiated with contact tracing, as the positive PCR test was clinically considered as persistent viral shedding (cases P6, P21, P23, and P24) without transmission capacity. This could pose a risk of onward transmission and nosocomial outbreaks, highlighting that serological tests should not be used to estimate the transmissibility period. Thus, collecting information about secondary transmission from reinfected cases could bring light on their transmission capacity and its impact on public health.

A high proportion of asymptomatic reinfections were identified through routine screening or contact tracing. A positive result for SARS-CoV-2 in an asymptomatic patient with a previous positive test could be wrongly interpreted as persistent viral shedding (especially in a context of scarce scientific evidence, such as the first months of the pandemic). Long viral shedding has already been reported, as well as intra-host viral evolution, but mainly in immunocompromised patients (Choudhary et al., 2021). Otherwise, new symptomatology arising in a previous positive case could be due to a COVID-19 reactivation, as has been described clinically and confirmed by sequencing (Chen et al., 2021; Lee J. T. T. et al., 2021; Pérez-Lago et al., 2021) occurring at a median of 57 days. In the present study, among confirmed reinfection cases with compatible symptoms in the second episode, two of them occurred as soon as 55 and 63 days (cases P9 and P16) after the first episode, but they were confirmed due to the higher number of mutations than the expected by the natural evolution of the virus. This higher number of mutations has already been observed in other cases of reinfections reported worldwide (Alshukairi et al., 2021; Lee J.-S. et al., 2021; Selhorst et al., 2021; Rahman et al., 2022), and some reports use it as evidence of reinfection (Sevillano et al., 2021; Tillett et al., 2021). On the other hand, cases P20 and P22 (occurring with an interval of 192 and 297 days, respectively) did not have a higher number of mutations than the expected. As they had compatible symptoms at reinfection and a history of recent contact, they were considered as true reinfections as per our previous settled criteria. Relying on the number of mutations between episodes is a good approach to distinguish between COVID-19 reinfection and reactivation (Choudhary et al., 2021). However, due to the low mutation rate of SARS-CoV-2, it remains challenging to distinguish reinfection from reactivation, especially in asymptomatic cases and with a short interval between episodes (Piri et al., 2021; Tang X. et al., 2021).

Despite the short time found between episodes in our study, none of them was classified as persistent viral shedding or reactivation. Thus, in a context of increased circulation of VOCs [related to higher transmissibility, such as alpha (B.1.1.7-like) and delta (B.1.617.2-like)], these results suggest considering a suspected case of reinfection for those presenting with a SARS-CoV-2 positive test and compatible symptoms in even less than 3 months after their first episode to proper implement prevention and control measures.

Observed variants in both episodes were similar to the sequences that circulated in the community at that moment (Hadfield et al., 2018; Andrés et al., 2021), providing evidence of being reinfections. Most of the mutations observed in the majority of reinfections in our study are either lineage-defining or have increased in prevalence due to their fitness-enhancing properties (Jacob et al., 2021; Troyano-Hernáez et al., 2021; Vilar and Isom, 2021). Despite mutations were identified throughout the whole genome, mutations in Spike protein were not overrepresented as could be expected because of a potential association with immune evasion. Noteworthy, some cases of confirmed reinfection did not present any mutation in the Spike protein. Moreover, most of these cases without mutations in the Spike did have a positive result in the serology test after the first episode as previously reported (Choudhary et al., 2021; Kulkarni et al., 2021), suggesting that these patients did not have an effective antibody response against a virus with similar antigenic properties to Wuhan strain.

Other mutations, such as those observed in nsp3, nsp6, and ORF10, are important to be further studied to clarify their implication in the virus’ fitness or in reinfection. The role of ORF10 is yet to be described, so it is difficult to hypothesize the reason why we found an association with the presence of symptomatology in reinfections. No relevant mutations related to immune evasion were found in the two hospitalized patients.

Finally, there is a lack of evidence on whether natural immunity to a non-VOC will have a good neutralizing capacity against VOCs. VOCs have proved to be more transmittable, which increases the concern of what effects these variants may have on public health, as they may lead to an increase in reinfections, and thus, in an extension in time of this pandemic. Hence, this article reinforces the need for continuous monitoring and sequencing of reinfections.

In addition, though no chronic active infections are reported in this manuscript, some cases have been observed in our hospital setting, mostly in immunocompromised patients (data not shown), and there are many publications reporting them. It is important to discern between reinfections and chronic infections, as the procedures applied are different in these cases, and it is also basic to differentiate when the virus is transmissible or not. In fact, there are some reports that postulate that these chronic infections are the origin of the emerging variants of SARS-CoV-2, such as the VOCs. Thus, monitoring these cases and studying those mutations occurring at the consensus level and those occurring at a low frequency are essential.

The findings in this study must be interpreted with caution as there are some limitations. First, the characteristics of the reinfected cases could be biased, as case identification in the present study was not performed by systematic surveillance. HCWs could be overrepresented as they have been a priority population for testing as well as hospitalized patients, as mild cases were mostly diagnosed by rapid tests performed in Primary Care Centers and these samples did not arrive at the hospital laboratory. Second, over 39 suspected reinfections, only 24 could be sequenced and confirmed, as WGS is suitable in cases with a moderate to high viral load.

## Conclusion

Reinfections can occur in a shorter time than previously reported, mostly in immunocompetent patients, and these episodes are more feasible to be less symptomatic and less severe than the first ones. This suggests that immune response was insufficient to prevent reinfection but modulated the clinical course in these cases. Moreover, reinfections are rare, but since they are usually asymptomatic, they may be underestimated and pose a risk for ongoing transmission. Thus, efforts should be addressed to strengthen reinfection SARS-CoV-2 surveillance. Performing WGS of all cases will allow us to rapidly identify key mutations and variants of special interest due to immune evasion or enhanced transmission.

## Data Availability Statement

The datasets presented in this study can be found in online repositories. The names of the repository/repositories and accession number(s) can be found in the article/supplementary material.

## Ethics Statement

The studies involving human participants were reviewed and approved by Comité de Ética de Investigación con medicamentos [Vall d’Hebron Institute of Research (VHIR)]. Written informed consent for participation was not required for this study in accordance with the national legislation and the institutional requirements.

## Author Contributions

MP, BB-B, CA, JE, MC, JQ, AA, and TP conceived the study. DG-C, AR, CCam, MGC, and SC-C participated in the design and implementation of the study. MP, CA, DG-C, CCam, and SC-C optimized the methodology of whole-genome sequencing. MM, CCas, KG-C, RV-M, and RM-M performed the whole-genome sequencing. AG-S, CA, and MP did the bioinformatic analyses. JE reviewed serological test results. OP-B, RZ, and BB-B reviewed medical records. MP and BB-B did the analysis of the results and drafted the manuscript that was critically reviewed by AA, MC, JQ, TP, and CA. All authors approved the final manuscript.

## Conflict of Interest

The authors declare that the research was conducted in the absence of any commercial or financial relationships that could be construed as a potential conflict of interest.

## Publisher’s Note

All claims expressed in this article are solely those of the authors and do not necessarily represent those of their affiliated organizations, or those of the publisher, the editors and the reviewers. Any product that may be evaluated in this article, or claim that may be made by its manufacturer, is not guaranteed or endorsed by the publisher.

## Footnotes

1
https://artic.network/ncov-2019
